# Low‐oxygen hormetic conditioning improves field performance of sterile insects by inducing beneficial plasticity

**DOI:** 10.1111/eva.13141

**Published:** 2020-11-04

**Authors:** Giancarlo López‐Martínez, James E. Carpenter, Stephen D. Hight, Daniel A. Hahn

**Affiliations:** ^1^ Department of Entomology and Nematology University of Florida Gainesville Florida USA; ^2^ Department of Biological Sciences North Dakota State University Fargo North Dakota USA; ^3^ USDA‐ARS Crop Protection and Management Research Unit Tifton Georgia USA; ^4^ USDA‐ARS Center for Medical, Agricultural, & Veterinary Entomology Tallahassee Florida USA

**Keywords:** anoxia, hormesis, modified atmospheres, sterile insect technique

## Abstract

As part of sterile insect technique (SIT) programs, irradiation can effectively induce sterility in insects by damaging germline genomic DNA. However, irradiation also induces other off‐target side effects that reduce the quality and performance of sterilized males, including the formation of damaging free radicals that can reduce sterile male performance. Thus, treatments that reduce off‐target effects of irradiation on male performance while maintaining sterility can improve the feasibility and economy of SIT programs. We previously found that inducing a form of rapid, beneficial plasticity with a 1‐hr anoxic‐conditioning period (physiological conditioning hormesis) prior to and during irradiation improves male field performance in the laboratory while maintaining sterility in males of the cactus moth, *Cactoblastis cactorum*. Here, we extend this work by testing the extent to which this beneficial plasticity may improve male field performance and longevity in the field. Based on capture rates after a series of mark release–recapture experiments, we found that anoxia‐conditioned irradiated moths were active in the field longer than their irradiated counterparts. In addition, anoxia‐conditioned moths were captured in traps that were farther away from the release site than unconditioned moths, suggesting greater dispersal. These data confirmed that beneficial plasticity induced by anoxia hormesis prior to irradiation led to lower postirradiation damage and increased flight performance and recapture duration under field conditions. We recommend greater consideration of beneficial plasticity responses in biological control programs and specifically the implementation of anoxia‐conditioning treatments applied prior to irradiation in area‐wide integrated pest management programs that use SIT.

## 1 INTRODUCTION

The sterile insect technique (SIT) is an ecologically friendly tool that can be successfully used in area‐wide integrative pest management programs. SIT uses ionizing radiation, such as gamma, X‐rays, and e‐beam radiation, to induce double‐stranded DNA breaks that cause dominant‐lethal mutations leading to sterility in insects. Sterile insects, usually males, are then released into a target area where these sterile males mate with wild females, thereby suppressing pest reproduction. SIT is used in several contexts from suppressing established pest populations to preventing the establishment of pests in areas of frequent introduction (Klassen & Curtis, 2005). Over the last 60 years, SIT has been successfully used to control invasions and outbreaks of multiple fly species (screwworms; *Cochliomyia hominivorax*, Mediterranean fruit flies; *Ceratitis capitata* and other tephritid flies) and moth species (pink bollworms; *Pectinophora gossypiella*, codling moths; *Cydia pomonella)* including the cactus moth; *Cactoblastis cactorum*) as part of area‐wide integrated pest management programs (AW‐IPM; Hight et al., 2005; Klassen & Curtis, 2005).

An important factor in successful SIT programs is choosing the right dose at which to irradiate the insects and doing so at the appropriate stage of development (age). Selecting a radiation dose is a fragile balance wherein one must expose insects to enough radiation to generate sufficient double‐stranded DNA breaks to cause the desired sterility, while avoiding undesirable negative side effects (Rull et al., 2012). Off‐target effects to irradiated insects can be caused by several types of damage; from direct ionization of critical cellular proteins, lipids, or other macromolecules to secondary downstream damage to macromolecules caused by the actions of free radicals that are produced as ionizing radiation splits gaseous oxygen and cellular water (Harman, 1956; Hulbert et al., 2007; von Sonntag, 1987).

The dose of radiation needed to ensure complete sterility may be considered too high if it leads to decreases in organismal performance: including reduced flight ability, mating, and longevity (Calkins & Parker, 2005; López‐Martínez et al., 2014; López‐Martínez & Hahn, 2012; Parker & Mehta, 2007). One solution is to use a radiation dose that causes the largest increase in male sterility, while promoting sexual competitiveness (Bloem et al., 1999, 2005). Most SIT programs tend to use the lowest possible dose that induces adequate sterility. However, when full sterility is too costly (i.e., insect competitiveness suffers and so does program efficacy), SIT programs can even use partial sterility with the goal of releasing better performing insects (reviewed by Carpenter et al., 2005).

SIT programs designed for lepidopterans (i.e., moths) use radiation doses that lead to partial parental sterility, a concept called inherited sterility (a.k.a., *F*_1_ sterility; North, 1975, Carpenter et al., 2001). This approach is used because lepidopterans are among the most radiation‐tolerant insects. Their high radiation tolerance is in part due to their holocentric chromosomal structures, which require very high ionizing radiation doses to cause enough double‐stranded DNA breaks to induce complete sterility (Bauer, 1967; Carpenter et al., 2005). However, the high doses needed to induce direct sterility affect moth performance negatively with side effects ranging from the inability to walk or fly well, to morphological deformations. Thus, to our knowledge, all active moth SIT programs use partial‐sterility approaches (Bloem et al., 2003; Carpenter et al., 2001; López‐Martínez et al., 2016). Using an inherited sterility approach to SIT requires an important balance between target dose and organismal performance. Integrating beneficial plasticity responses to increase irradiated male performance without decreasing male infertility would be particularly useful for implementation in lepidopteran SIT programs.

The use of low oxygen during irradiation was first studied in lepidopterans more than 40 years ago, where it was noticed that nitrogen atmospheres had protective effects on moth performance (Robinson, 1975). Since then, irradiation in modified (low‐oxygen) atmospheres has been actively used in fruit fly SIT programs, but this approach has not been applied to moth SIT programs despite the evidence of its effectiveness (Bakri et al., 2005; FAO/IAEA/USDA, 2003). More recently, work from our group has shown that anoxia conditioning prior and during irradiation improves multiple metrics of organismal performance (treatment survival, flight ability, mating, and longevity) in laboratory assays in flies (López‐Martínez & Hahn, 2012, 2014; Teets et al., 2019) and moths (López‐Martínez et al., 2014, 2016). It has been suggested that this type of beneficial plasticity application in insect systems might be the kind of powerful tool needed for improving quality in SIT (Sørensen et al., 2012). It is well recognized that environmental temperature can affect the success of sterile male releases (Bloem et al., 2005; Boersma et al., 2019; Sørensen et al., 2012). Beneficial acclimation to low temperatures has already been shown to improve flight performance and recapture rates of male coddling moths on cool spring days (Chidawanyika & Terblanche, 2011), and such promising results of beneficial acclimation regimes should be investigated for other stresses that may affect sterile male performance.

Here, we extend our previous laboratory‐based work on anoxia‐conditioning treatments by applying this technique to a series of field mark release–recapture experiments. Male moths treated to 1 hr of anoxia conditioning prior to and during irradiation were found to be more active in the field than unconditioned moths irradiated in normoxia by all three of our measures of performance: day of capture after release, distance traveled, and direction of capture. Anoxia‐conditioned irradiated males were trapped in the field longer than nonconditioned irradiated males. Additionally, anoxia‐conditioned males were captured farther away from the release point and over a wider range of our trapping array. Our data indicate that inducing beneficial plasticity by anoxia‐conditioning males prior to irradiation, a treatment that was previously shown to reduce postirradiation oxidative damage and increase performance in the laboratory (López‐Martínez et al., 2014; López‐Martínez, et al., 2016) also increases performance and longevity in the field.

## 2 MATERIALS AND METHODS

### 2.1 Animal preparation

All cactus moths, *Cactoblastis cactorum* (Berg) (Lepidoptera: Pyralidae), used in these experiments were reared at the USDA‐ARS Crop Protection and Management Research Unit in Tifton, GA. This is the same colony that was used for cactus moth SIT releases as part of a binational cactus moth eradication program between the United States and Mexico (Hight et al., 2005). Newly emerged adult cactus moths were sorted every morning in a 4°C room into 100 × 15‐mm petri dishes, kept immobilized at 4°C to prevent wing damage due to movement inside the petri dishes, and transported from USDA‐Tifton to the University of Florida in Gainesville, FL (250 km away). At the University of Florida, they were once again sexed and sorted into groups for treatments. Moths were kept at a relatively low density in the petri dishes (100–150 moths < 1.5 cm in length) for each treatment and release.

### 2.2 Radiation treatments

Male adult moths were irradiated within 24 hr. of adult emergence at 200 Gy using a Varian L‐1000A electron‐beam irradiator (5.2 MeV, 1.5 kW, CGR MeV, France) with a copper plate to convert electron‐beam radiation into X‐rays at the Florida Accelerator Services and Technology facility within the Division of Plant Industry of the Florida Department of Agriculture and Consumer Services, Gainesville, Florida.

Moths were exposed to one of two treatments: normoxia (normal air) and irradiation at 200 Gy (Nx200), the current SIT dose treatment for cactus moth inherited sterility, or 1 hr of exposure to anoxia (<0.1% oxygen) followed immediately by irradiation at 200 Gy while still in anoxia (Ax200). Moths were held in 100 × 15 mm petri dishes during irradiation and kept at 3.5 to 4.5°C using 30 × 30 cm cold blocks. All petri dishes were bagged and sealed in custom‐sized 4‐mil‐thick polypropylene bags. Normoxia moths were sealed in bags that had been heavily perforated with a 20‐gauge syringe to allow the flow of air into the dishes containing moths, while anoxia‐conditioned moths were sealed in intact bags flushed with nitrogen as previously described to induce a hormetic antioxidant response for this species (López‐Martínez et al., 2014). Gafchromic HD‐810 film (International Specialty Products) was used to verify the dose and uniformity of radiation received by the moths by placing film strips inside of paper envelopes at the top and bottom of the petri dishes prior to irradiation. Our dose uniformity rate (DUR) for our release experiments was 1.01.

After irradiation but prior to field release, moths were marked with fluorescent powder (DayGlo Color Corp). An array of different colors was used to differentiate moths between treatments (normoxia‐treated versus. anoxia‐conditioned) and between subsequent releases (orange, green, red, blue, or yellow). These powders have been found not to be detrimental to moth performance (Hagler & Jackson, 2001). The colors used were alternated weekly between the treatments to prevent color bias in our handling (or predation). Marked moths were identifiable under an UV light source for their full lifespan in the laboratory (3 weeks) and field (~1 week). We also noted that even after dead and stored at room temperature (~25°C), the color was still present at 1 month.

### 2.3 Laboratory flight assays

Throughout the field mark–release–recapture experiments across different seasons, male moths were randomly chosen after irradiation and subjected to our laboratory‐based flight ability assay as a metric of quality consistency in moth performance. Briefly, three to five groups of 10 moths each were chosen from the treated individuals, prior to marking, and tested. For each test, a single moth was gently flung from a petri dish (100 × 15 mm) in a darkened 3 × 6 m room with a red light on to observe moths. Moths were scored as either flying or dropping to the ground. This flight propensity assay was taken directly from our previous work on anoxic conditioning and performance in the laboratory (López‐Martínez et al., 2014). In our previous study, propensity to fly within a treatment was also strongly positively associated with greater flight durations and flight distances among moths in that treatment. At least one flight ability test was carried out during each field‐release trial; haphazardly either early, mid‐, or late in the trial to act as a quality control check.

### 2.4 Field mark–release–recapture experiments

Irradiated moths were transported at 4°C to the University of Florida's Plant Science Research and Education Unit (PSREU) facility in Citra, Florida (38 km south of the University of Florida), to carry out the release–capture experiments. PSREU has a large section of the facility dedicated to organic farm research, including pesticide‐free zones. Within one of these areas, the Florida Department of Agriculture and Consumer Services (FDACS) maintains a prickly pear cactus (*Opuntia* sp.) plot measuring roughly 50 by 25 meters specifically for cactus moth research (Hight & Carpenter, 2016). Field releases were carried out in the afternoons between 3:30 p.m. and 5 p.m., a time coinciding with the peak of male cactus moth flight activity (Sarvary et al., 2008).

Our first field‐release study was performed in mid‐summer from July 31, 2012, to August 9, 2012. The average temperature at the time of the first release (~3:30 to 4:30 p.m.) was 30.4°C with an average daily high of 32.1°C and an average nightly low of 22.5°C. We also include additional information on wind speed, precipitation, and cloud cover during all three releases in a Figure S1. The cactus patch, measuring ~1,250 m^2^ in area, was crossed with seven transects running east to west. Each transect contained nine heavy‐duty steel posts that were equally spaced along each transect. A Pherocon 1C wing trap (Trécé Incorporated) sat at a height of 152.4 cm on top of each steel post (total of 63 traps). Pherocon traps have a flexible plastic top and a plastic bottom which is coated with a sticky material on the inside that traps the moths that enter the trap. Synthetic female sex pheromone baits (Scentry Biologicals) pinned to the top inside lid of the trap attracted males into the trap. The pheromone bait contained cactus moth pheromone compounds previously isolated by Heath et al. (2006). Steel wire was used to secure the pheromone traps to the post.

In the first week of the trial, an average of 150 male moths per treatment was released from the center of the trapping area every day for four days (7/31–8/3/2012). The following week (8/6–8/9/2012), an average of 340 male moths was released per treatment on each of 4 days. Pherocon traps were checked every morning (9a.m.) during releases and for 1‐week after releases ended. Traps were checked in the morning to distinguish moths from a previous release from those being released on that day. Traps that contained cactus moths or other insects were removed and replaced daily and then taken back to the laboratory for treatment verification and counting.

**Figure 1:**
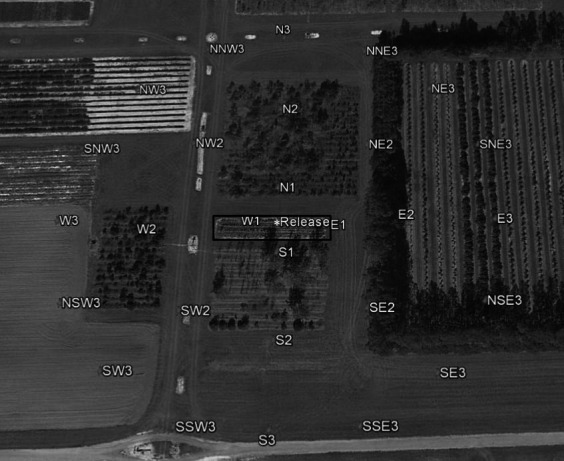
The mark release–recapture site at the University of Florida's Plant Science Research and Education Unit (PSREU) facility in Citra, Florida, identifying field experiment trapping areas. The black rectangle marks the trapping area for experiment 1 consisting of 1,250 m^2^ with seven transects containing nine traps each. The 28 traps in the larger 28,350‐m^2^ area were the design for experiments 2 and 3. The release point for all trials is marked with an asterisk in the center. Photo credit: Google Earth

Our original trap capture area was designed based on previous studies that showed such an area was adequate to capture a large proportion of irradiated cactus moths that were released (Hight & Carpenter, 2016). However, we noticed that we captured very few anoxia‐conditioned moths and that they were mostly captured in the outermost traps in the grid; our first evidence that anoxia conditioning affected moth field performance. Based on this observation, we redesigned the trapping area and increased it to ~28,350 m^2^ (~23 times larger) for subsequent studies. The traps were set in outward concentric circles from the center point of release at 15, 55, and 95 m. The traps were placed using cardinal coordinate orientation. There were four traps at 15 m from the release point (N, S, E, W), eight traps at 55 m (N, NE, E, SE, S, SW, W, NW), and 16 traps at 95 m (N, NNE, NE, ENE, E, ESE, SE, SSE, S, SSW, SW, WSW, W, WNW, NW, NNW) (Figure 1) for a total of 28 traps. One aspect we did not quantify in our first trial was how many days after release were the moths being captured. To this end, in subsequent trials we switched from daily releases to releases every 2–4 days, alternating marking colors to assess possible field performance‐related longevity.

Our second field trial ran in the fall from 10/15/2012 to 11/6/2012. Male moths were released twice a week for 3 weeks. The first week (10/15/2012 and 10/18/2012), 198 moths were released per treatment. The second week (10/22/2012 and 10/25/2012), ~189 moths were released per treatment, and in the third week (11/1/2012 and 11/5/2012), ~312 moths were released per treatment. As in our first trial, traps were checked every day in the morning during the experimental period (10/15/2012 to 11/6/2012), continuing daily throughout the week that followed the last release date. The average temperature at the time of the second release (~3:30 to 4:30 p.m.) was 26.8°C with an average daily high of 27.3°C and an average nightly low of 14.7°C (additional weather information is available in Figure S1).

We ran a third mark–release–recapture trial in the spring of 2013. This third field experiment used the same field plot/trap design as the second field trial. The third trial ran for a month in the late spring (5/7/2013–6/4/2013). The first week (5/7/2013 and 5/9/2013) 634 and 735 moths were released per treatment. The second week (5/14/2013 and 5/16/2013) 782 and 768 moths were released. The third week (5/21/2013 and 5/23/2013) 721 and 823 moths were released, and in the final week (5/29/2013 and 5/31/2013), 591 and 625 moths were released. As in previous trials, traps were checked daily and replaced (when necessary) including during the week after the last release. The average temperature at the time of the third release (~3:30 to 4:30 p.m.) was 27.6°C with an average daily high of 29.1°C and an average nightly low of 15.5°C (additional weather information is available in Figure S1).

### 2.5 Statistical analyses

Because laboratory flight assays data met the assumptions of normality and homoscedasticity of variances, they were analyzed using two‐way ANOVA with treatment (normoxia‐treated or anoxia‐conditioned), field trial (first, second, or third), and their interaction (treatment * trial) as factors. Our first field trial consisted of four replicate releases of 150 moths per treatment and a replicated experiment consisting of four replicate releases of 340 moths. Our second field trial consisted of six sequential releases spread out over 3 weeks and ranging from 189 to 312 moths per release per treatment. Our third and final trial consisted of eight sequential releases over the course of a month, ranging from 591 to 823 moths per treatment. Overall recapture rate within each field trial was analyzed using a general linear model with gamma (1st trail), Poisson (2nd trial), log normal distributions, and treatment (normoxia‐treated or anoxia‐conditioned) as a factor. To test the extent to which anoxia‐conditioned moths might be captured more than normoxia‐treated moths as time since release increased, we used a general linear model with gamma Poisson distributions and treatment (normoxia‐treated or anoxia‐conditioned) as well as day since release, and their interaction (treatment * day) as factors. To see whether local weather conditions differed among the trials, we downloaded the following from the Weather.com archive for the nearest reporting station with available data for our field site (zip code 32113): temperature in the afternoon at 4:00 p.m., maximum daily temperature, minimum daily temperature, wind speed at 4:00 p.m. in the afternoon, maximum daily wind speed, and percent day time cloud cover. One‐way ANOVAs were used to test for differences in afternoon temperature, maximum daily temperature, afternoon wind speed, maximum daily wind speed, and cloud cover with separation of means done by Tukey's post hoc correction for multiple comparisons. Minimum daily temperature data did not meet the assumption of heterogeneity of variances. Trials 2 and 3 which were held in fall and spring, respectively, had much more nightly temperature variation than the trial 1 that was held in late summer, so differences among 3 trials in minimum nightly temperatures were assessed with a nonparametric, rank‐sum test (Kruskal–Wallis). GLMs, ANOVAs, and the Kruskal–Wallis test were performed in JMP 15 (SAS software, Raleigh NC). Directional recapture data were analyzed using Oriana 4 circular statistics analysis software (Kovach Computing Services, Isle of Anglesey, UK). Rayleigh tests were carried out in this software package to test if the distribution of the recaptures was random or directional.

## 3 RESULTS

### 3.1 Laboratory flight assays

**Figure 2:**
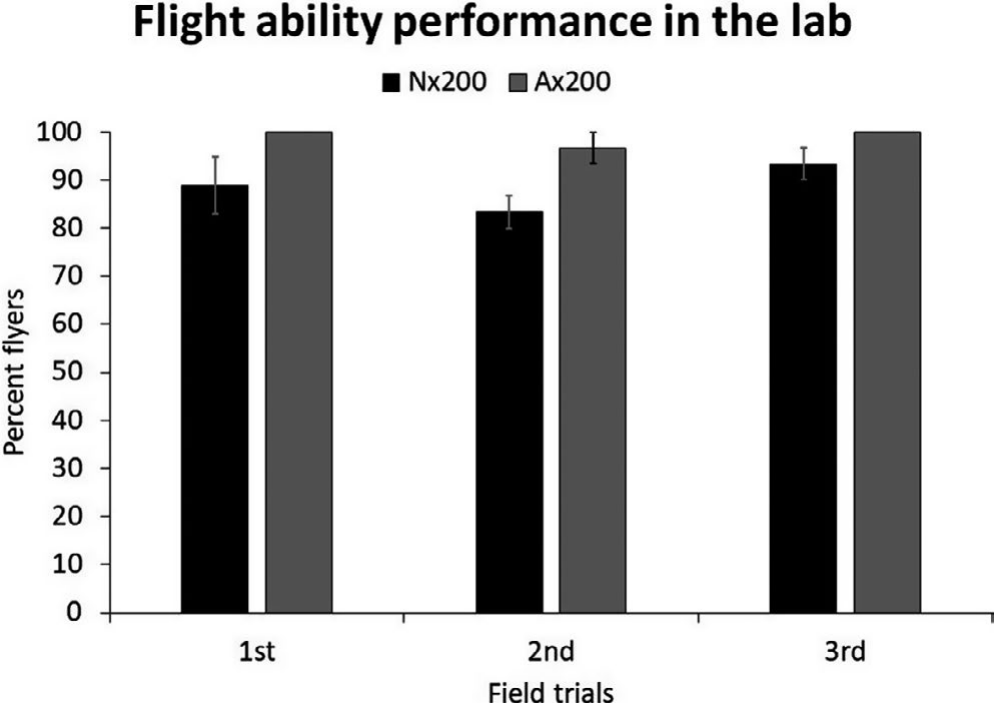
Proportion of moths that flew in bioassays after being irradiated at 200 Gy and treated with one of two modified atmospheres, normoxia (Nx200) or anoxia (Ax200), in each of three performance trails. Although anoxia‐hormesis moths had greater flight ability in laboratory assays, flight ability was high (>85%) across both treatments in all three release trials. Periodic flight ability trails were carried out on haphazardly selected groups of moths as a quality control measure throughout the field releases for trials 1, 2, and 3. Means and standard errors are shown but note that the standard error is so small for the hormetic‐treated group in the first and third trail that it does not show in the figure

Overall, anoxia‐irradiated moths were more likely to fly than those irradiated in normoxia without conditioning across all three trials (Figure 2; ANOVA, *F*_5,12_ = 3.213, *p*_full model_ = .0453; *F*_1,1_ = 11.456, *p*_treatment_ = .005; *F*_2,2_ = 1.83, *p*_trial_ = .202; *F*_2,2_ = 0.475, *p*_treatment * trial_ = .633). This served as a confirmation of previous work where multiple metrics of laboratory flight performance (flight propensity, duration, and distance) were improved by anoxia hormesis (López‐Martínez et al., 2014). Additionally, laboratory flight tests served to verify that moth quality after treatment was consistently high among our three release trials with >80% of moths flying in all treatments.

### 3.2 Field mark–release–recapture

**Figure 3:**
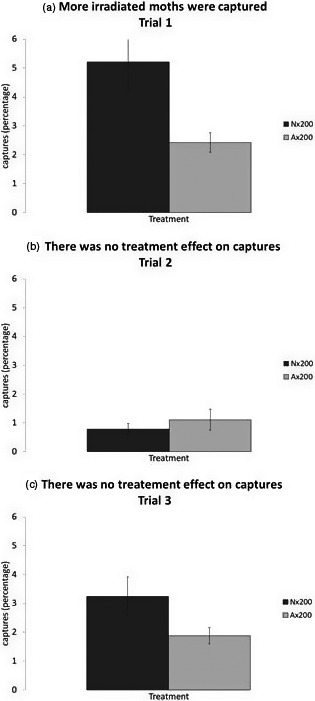
The overall number of captured male moths released after being irradiated at 200 Gy and treated with one of two modified atmospheres, normoxia (Nx200) or anoxia (Ax200), in each of three performance trials; the number of captured males was variable among trials 1 (a), 2 (b), and 3 (c). Means and standard errors are shown on graph

Our first field trial with the 1,250‐m^2^ plot had an overall average capture rate of 3.82 ± 1.04%. The average capture rate for the male moths irradiated in normoxia (Nx200, 5.21 ± 1.04%) was 115% higher than the average for the anoxia‐irradiated males (2.42 ± 0.34%, Figure 3a, GLM, *χ* ^2^ = 4.314, *df* = 1, *p* = .0378). The anoxia‐irradiated moths that we captured were predominantly recorded in the outermost eastern traps in our plot. We used this observation in the decision to expand plot size for subsequent field releases.

**Figure 4:**
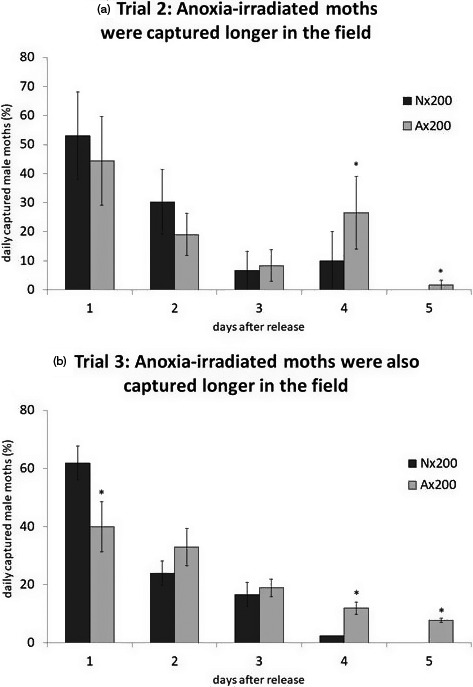
A higher number of anoxia‐irradiated (Ax200) male moths were captured four and five days after the original release than normoxia‐irradiated (Nx200) male moths in both trial 2 (a, *p* = .025) and in trial 3 (b, *p* < .001). For trial 3, the proportion of male moths captured for five days after release was different between treatments. More than half Nx200 moths were captured on day 1 while Ax200 moth captures were spread out and these moths were still captured on day 5 in both trials. Means and standard errors are shown on graph. Asterisks indicate a significant difference in trap captures between Ax200 and Nx200 moths on that particular day

Our second field trial with a trap area of 28,350 m^2^ had a lower capture rate than the pilot study at 0.94 ± 0.21%. With our new, larger trapping area overall we captured approximately 42% more anoxia‐conditioned moths (1.11 ± 0.36% capture) than normoxia‐treated moths (0.78 ± 0.2% capture), but this higher capture rate was not significantly different (Figure 3b, GLM, *χ* ^2^ = 1.594, *df* = 1, *p* = .2067). When breaking down our results by daily captures, more anoxia‐conditioned moths than normoxia‐treated moths were trapped on days 4 and 5 after their initial release (Figure 4a, GLM, *χ* ^2^_full model_ = 55.746, *df*_full model_ = 9, *p*_full model_ < .0001, *χ* ^2^_treatment_ = 1.594, *df*_treatment_ = 1, *p*_treatment_ = .207, *χ* ^2^_day_ = 38.595, *df*_day_ = 4, *p*_day_ < .0001, *χ* ^2^_treatment*day_ = 13.726, *df*_treatment*day_ = 4, *p*_treatment*day_ = .0082). These data indicated that anoxia‐conditioned moths were living longer, being more active through time, or a combination of both, after being released into the field. The sample size of captures in this second trial was too small to allow for an adequate comparison of the grid captures over distances.

**Figure 5:**
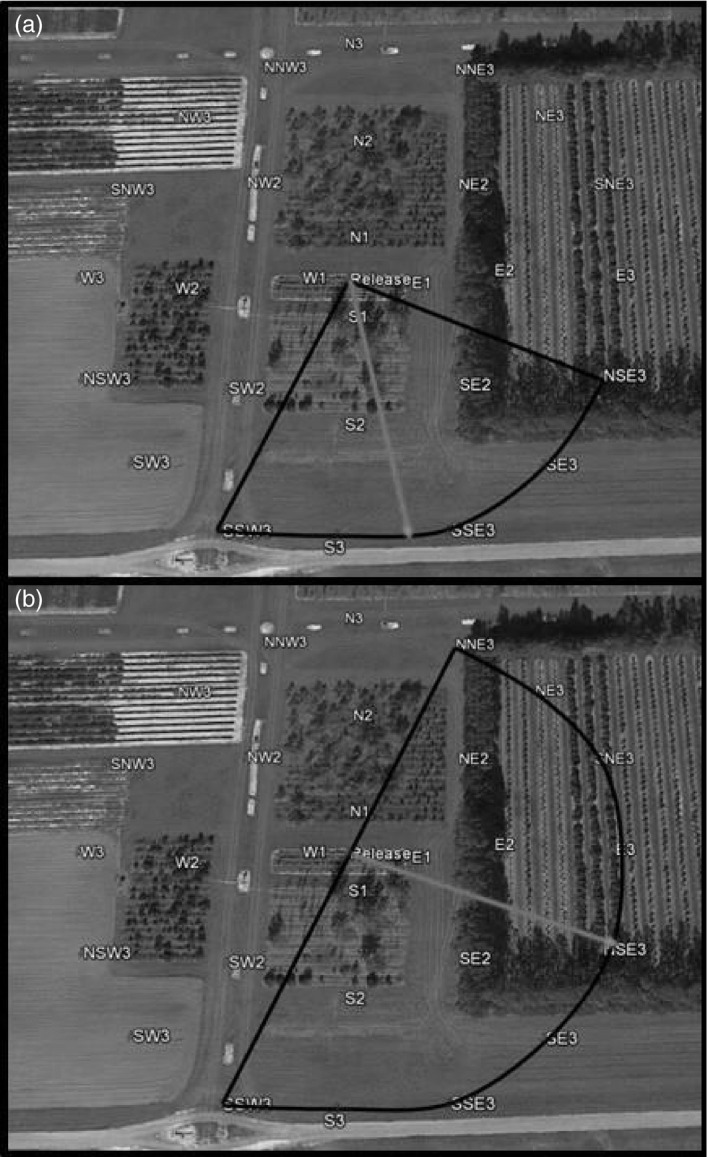
The mark release–recapture site at the University of Florida's Plant Science Research and Education Unit (PSREU) facility in Citra, Florida, identifying the area were most normoxia‐irradiated moths (Nx200) were captured (a) and most anoxia‐irradiated moths (Ax200) were captured (b). Normoxia‐irradiated moths were captured in greater proportion in a smaller section of the overall trap grid (a), while anoxia‐irradiated moths were captured over a much larger area (b). Photo credit: Google Earth

The third and last field trial performed in spring 2013 had a capture rate of 2.56 ± 0.4%. In this trial, we captured more normoxia‐treated moths than anoxia‐conditioned moths, but this difference was not significant (Figure 3c, GLM, *χ* ^2^ = 3.396, *df* = 1, *p* = .0653). Specifically, we captured approximately 72% more normoxia‐treated moths (3.24 ± 0.69% capture) than anoxia‐conditioned moths (0.78 ± 0.2% capture). When breaking down our results by daily captures, as in the second field trial, anoxia‐conditioned moths were captured more frequently in days 4 and 5 (Figure 4b, GLM, *χ* ^2^_full model_ = 330.381, *df*_full model_ = 9, *p*_full model_ < .0001, *χ* ^2^_treatment_ = 38.904, *df*_treatment_ = 1, *p*_treatment_ < .0001, *χ* ^2^_day_ = 229.987, *df*_day_ = 4, *p*_day_ < .0001, *χ* ^2^_treatment*day_ = 25.676, *df*_treatment*day_ = 4, *p*_treatment*day_ < .0001). The normoxia‐treated group shows a circular–linear correlation (*r* = 0.251, *p* < .0001) meaning that the distance traveled by the moths is related to the direction of that travel. The normoxia‐treated moths that traveled ≥ 15 m did so in a southeasterly direction. The anoxia‐conditioned moths in contrast showed no circular–linear correlation (*r* = 0.141, *p* = .129), indicating that these moths traveled distances uniformly distributed in all directions. Normoxia‐treated moths did not have a uniform distribution (Rayleigh test *Z* = 4.188, *p* = .015) with a south–southeast vector mean (*µ* = 165.08°) and *r* = 0.149 (mean vector length). Anoxia‐conditioned moths had a uniform distribution (*Z* = 0.891, *p* = .41) with an east‐southeasterly vector mean (*µ* = 108.94°) and *r* = 0.092 (mean vector length). These results showed that normoxia‐treated moths flew in a directed pattern along 165°, while anoxia‐conditioned moths flew more uniformly across the capture area. The mean vector length (*r*) revealed that a higher concentration of the normoxia‐treated moths was captured around the south–southeastern vector versus a lower concentration of the anoxia‐conditioned moths were captured along their east‐southeasterly vector. An overall comparison of the grid captures over distances indicated that there was a difference in the number of captures between treatments (*χ* ^2^ = 124.34, *p* < .0001). Normoxia‐treated moths were captured more often in the 15‐ and 55‐m traps while anoxia‐conditioned moths were captured more often in the 55‐ and 95‐m traps. Normoxia‐treated moths (Figure 5a) were mostly captured in a smaller fraction of the trap grid that faced the southeastern region of the trap zone closer to the release point while anoxia‐conditioned moths were captured across most of the eastern portion of the trapping range and farther from the release point (Figure 5b). Additionally, more than half (~60%) of the normoxia‐treated moths were captured the day after the initial release while it took two days to capture that many anoxia‐conditioned moths (Figure 5; *χ* ^2^_day_ = 229.987, *df*_day_ = 4, *p*_day_ < .0001).

### 3.3 Weather across trials

Recapture rates were substantially lower during our second trial, which was held in late fall, than our first and third trials that were held in late summer and late spring, respectively. Thus, we tested whether the second trial had lower temperatures or clear differences in other weather factors that might have affected recapture rates. With respect to temperature, there were no differences in afternoon temperatures among the three trials (Figure S1a, ANOVA, *F*_2,21_ = 2.74, *p* = .09), but both daily maximum temperatures and daily low temperatures were significantly higher in the first trial held in late summer than in either the second or third trial, which did not differ from each other (Figure S1b, ANOVA, *F*_2,21_ = 8.15, *p* = .003, and Figure S1c, Kruskal–Wallis, *χ* ^2^ = 14.9, *df* = 2, *p* < .001, respectively). There were no differences among the three trials in afternoon wind speeds (Figure S1d, ANOVA, *F*_2,21_ = 1.49, *p* = .25), maximum daily wind speed (Figure S1e, ANOVA, *F*_2,21_ = 0.48, *p* = .63), or cloud cover (Figure S1f, ANOVA, *F*_2,21_ = 0.13, *p* = .87). No rainfall was recorded during any of the trials.

## 4 DISCUSSION

In our second and third field trials, with the larger trapping area, we showed that inducing a beneficial plastic response with anoxia conditioning improved both the dispersal distance and capture duration of irradiated sterile males in the field. Anoxia‐conditioned moths dispersed farther than normoxia‐treated moths in our trap array with most anoxia‐conditioned moths captured in the outermost two concentric trap circles (55 and 95 m), whereas normoxia‐treated moths were mostly captured in the closest two trap circles (15 and 55 m). Anoxia‐conditioned male moths were also captured over a larger area (~14,000 m^2^; Figure 5b), while unconditioned, irradiated male moths were captured in an area roughly half the size (~7,000 m^2^; Figure 5a). Capturing more anoxia‐conditioned males over an area this large supports our assertion from the first field trial that our poor capture of anoxia‐conditioned moths was likely due to them flying beyond the original, smaller 1,250‐m^2^ grid whereas many unconditioned sterile male moths were captured in this smaller area.

In addition to dispersing farther, anoxia‐conditioned moths were also captured over a wider area of our sampling grid. Most of the unconditioned moths dispersed into an area to the south–southeast of the release area (Figure 5a), whereas anoxia‐conditioned moths were captured over a broader area encompassing the south to east (Figure 5b). Wind patterns at our field site blow largely to the south and southeast and are strongest in the late afternoon (2–4 p.m., Florida Automated Weather Network, University of Florida), potentially driving dispersal in this direction in both normoxia‐treated and anoxia‐conditioned moths. However, anoxia‐conditioned moths also dispersed more frequently to the east into an area that includes an organic orange grove and a windbreak of trees that we think may provide better shelter than the open mowed fields to the south. We believe that there is a strong need for released moths to find shelter both from warm, desiccating conditions in full sunlight and from predators, including birds and dragonflies that occur in our release site. We observed birds swooping down to take moths just after release, and predation is a major factor affecting field‐released sterile moths and flies (Hendrichs & Hendrichs, 1998; Iwahashi, 1976; Schroeder et al., 1973). These field data showing differences in dispersal distance reinforce a previous laboratory study that showed higher levels of flight performance in anoxia‐conditioned males than normoxia‐treated sterile male cactus moths; specifically, anoxia‐conditioned male moths had a highest propensity of flight and flew further over a longer period of time than normoxia‐treated male moths (López‐Martínez et al., 2014).

Beyond improving dispersal, anoxia‐conditioned sterile male moths were also captured for a longer period of time in the field in our last two field experiments where we collected daily recapture data (Figure 4). While the daily capture rate decreased over time for both groups, more anoxia‐conditioned sterile males were captured on days four and five than unconditioned irradiated males, and no males were captured more than five days after release. This observation of longer duration of capture in the field suggests that anoxia conditioning extends the effective period for sterile males in the field. These data are also consistent with previous laboratory experiments showing that anoxia conditioning increases longevity in the laboratory, reinforcing the idea that testing performance‐enhancing treatments in laboratory‐based assays can predict field performance. Survival in the field dictates how often sterile male releases must occur, therefore influencing cost (Hendrichs et al., 2005; Lance & McInnis, 2005), and because mass‐rearing selects for short‐lived individuals (Cayol, 2000), improving longevity can have wide implications for control. Additionally, mating competitiveness is normally reduced as a consequence of mass‐rearing, and any improvements to sterile males must take into account the duration of effectiveness in the field, in addition to improving mating (Meats, 1998). The same beneficial plasticity anoxia treatment that increases duration of recapture in the field also increases mating competitiveness compared to normoxia‐treated males in a previous laboratory study (López‐Martínez et al., 2014). Thus, the effects of anoxia conditioning have potential for improving two shortcomings of SIT applications (Meats, 1998). This improved performance data aligns with predictive models aimed at optimization of sterility while preserving performance (Meats, 1998; Parker & Mehta, 2007), and without the need to lower the irradiation dose any further, anoxia conditioning can improve sterile male moth performance.

An important observation is that recapture rates varied dramatically among our three field trials (3.82 ± 1.04%, 0.94 ± 0.21%, and 2.56 ± 0.4% overall, respectively). Why the recapture rate was so much lower in our second trial compared to our first and third trials is unclear because the laboratory flight assays suggested no major deficit in the overall quality of insects used in the second release (>80% fliers, Figure 2). Environmental factors including temperature, winds, and precipitation can affect sterile moth releases (Bloem et al., 2005; Boersma et al., 2019; Chidawanyika & Terblanche, 2011; Sørensen et al., 2012). Our second field trial was done in the late fall (October 15–November 6, 2012) whereas our first release was done in late summer (July 31–August 9, 2012) and our third release was done in late spring (May 7–June 4, 2013). However, our analyses of weather data showed no clear pattern for why we captured fewer moths in the second trial because while daily high and daily low temperatures were higher in the first release than in the second release, there were no significant differences in weather parameters between the second and third release, and recapture rates were relatively high in the third release. We could speculate about factors that may have affected the second release more than the first and third releases leading to lower recaptures, from greater predation in the fall to pesticide drift from agriculture fields outside of our field site, but we have no clear evidence for why recapture rates were lower in the second release and must ascribe this to unexplained field variation.

Even though the benefits of anoxia (Ashraf et al., 1975; Fisher, 1997; Robinson, 1975) and other low‐oxygen treatments (Hooper, 1971; Nestel et al., 2007; Ohinata et al., 1977) on irradiation and performance in an SIT context have been known since the 1970s, it was just in the last 10 years that some of the mechanisms behind this type of beneficial plasticity have been elucidated. This protective response to anoxia conditioning is partially rooted in a conserved mechanism described by the preparation for oxidative stress hypothesis (Giraud‐Billoud et al., 2019; Hermes‐Lima et al., 1998), where mitochondria that experience low oxygen prepare for reperfusion by elevating antioxidant defenses. In a previous laboratory study, we found that anoxia conditioning triggered increases in total antioxidant capacity just after treatment in male cactus moths, and decreased oxidative damage to both proteins and lipids was still decreased 5 days after irradiation in anoxia‐conditioned male cactus moths compared to normoxia‐treated males (López‐Martínez et al., 2014). Similarly, in a tephritid fruit fly system, we previously found that the activity of multiple antioxidant enzymes was increased for at least 24hrs after anoxia conditioning, which was associated with a decrease in postirradiation oxidative damage as long as 10 days after irradiation (López‐Martínez & Hahn, 2012). Together these studies demonstrate that enhancing antioxidant capacity even transiently at the time of irradiation can have long‐lasting positive effects on both oxidative damage and sterile male performance. The mechanisms behind anoxia hormesis (Calabrese et al., 2007) are likely multifarious and involve other biochemical and cellular responses in addition to antioxidants (Berry & López‐Martínez, 2020; Harrison et al., 2018, Berry and López‐Martínez unpublished data). However, the importance of antioxidant capacity in preserving sterile male performance after irradiation was recently reinforced by a study that specifically overexpressed the primary mitochondrial antioxidant enzyme, superoxide dismutase (SOD), and showed increased mating and a reduction in accumulated damage after irradiation of sterile males in a tephritid fruit fly pest that was not anoxia‐conditioned (Teets et al., 2019).

The early work showing hypoxia improved sterile male performance in tephritid fruit flies was compelling enough for many fruit fly SIT facilities worldwide to implement the use of hypoxia in their protocols (Bakri et al., 2005; Calkins & Parker, 2005; Nestel et al., 2007). The ample work showing that hypoxia improves mating competitiveness in fruit flies (Ashraf et al., 1975; Hooper, 1971; Ohinata et al., 1977) has translated into the widespread use of oxygen manipulation in fruit fly programs, but the implementation of anoxia has been lagging in moth SIT programs. The fact that our anoxia‐conditioned moths were recaptured for longer after a field release shows the potential of this type of beneficial plasticity application to the economy of pest control. The Canadian Okanagan‐Kootenay Sterile Insect Release (OK SIR) program is the longest running sterile insect release program for the codling moth, *Cydia pomonella* L. (Lepidoptera: Tortricidae), at a current annual cost of C$3.7 million (Thistlewood & Judd, 2019). The use of anoxia conditioning in this program (i.e., having moths effective longer in the field) could lead to a reduction in the numbers of moths being released and/or a reduction in the total number of releases required for pest suppression annually. Other lepidopteran SIT programs include the false codling moth *Thaumatotibia leucotreta* (Meyrick) (Lepidoptera: Tortricidae) in South Africa, pink bollworm *Pectinophora gossypiella* (Saunders) (Lepidoptera: Gelechiidae) in the United States and Mexico, and cactus moth in the United States and Mexico (Marec & Vreysen, 2019). Economic losses and the cost of treatment are in the millions of dollars across each program and the potential of anoxia conditioning to lower economic losses while reducing the cost of treatment is the very reason this type of beneficial plasticity should be widely considered for implementation.

The benefits of anoxia conditioning extend beyond improving the performance of animals in the field and into the potential for lowering the cost to control lepidopteran pests globally. The application of low‐oxygen pretreatments is straightforward and frequently yields similar protective results across different systems (Berry & López‐Martínez, 2020). Beyond the manipulation of oxygen, other types of beneficial plastic responses have been tested, such as temperature conditioning. Temperature conditioning improves flight performance in the false codling moth (Boersma et al., 2019). Given our connection between improved flight performance and duration of capture in the field (López‐Martínez et al., 2014), it is likely that temperature conditioning has potential synergy with anoxia conditioning. We can envision SIT workflows where multiple hormetic treatments could be applied concurrently to have even greater positive effects on the performance of sterile males and even greater cost savings in SIT programs. In addition to SIT, anoxia conditioning may improve mating competitiveness and extend effective duration in the field for biological control agents, a big component of integrated pest management (Sørensen et al., 2012). In summary, we believe that the implementation of anoxia conditioning in active moth SIT programs would have positive effects on male field performance potentially including higher efficacy at suppressing pest populations and potential cost savings.

## CONFLICT OF INTEREST

None declared.

## Supporting information

Figure S1

## Data Availability

Data for this study are available: https://doi.org/10.5061/dryad.vx0k6djpn
