# Supplementary figures and images for: Low‐oxygen hormetic conditioning improves field performance of sterile insects by inducing beneficial plasticity
Source: Evol Appl. 2020 Nov 4;14(2):566–76. doi: 10.1111/eva.13141 (PMC7896707; doi:10.1111/eva.13141)

**Fig. S1**

**
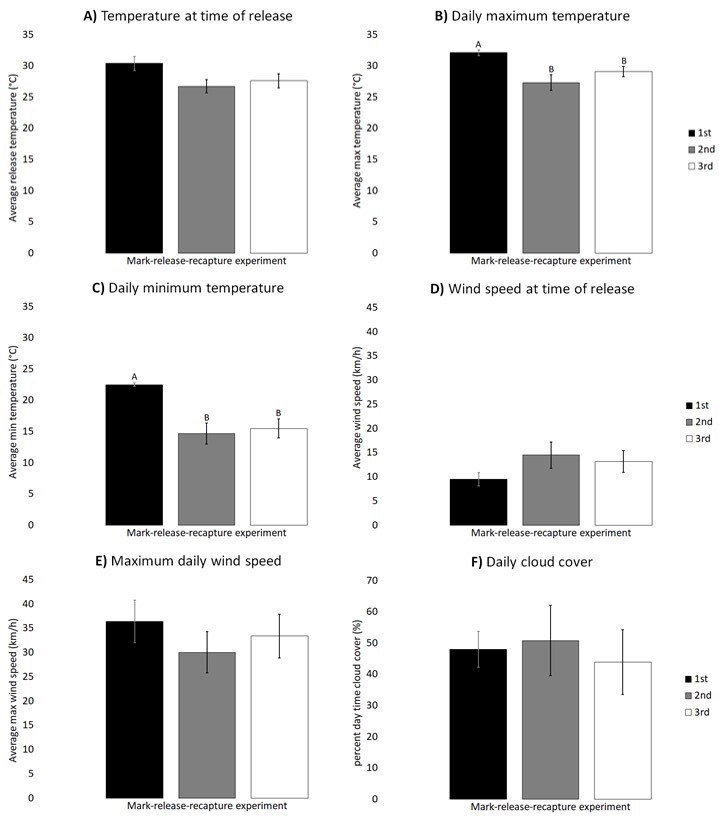
**

Supplement: Supplementary file 1 — Figure S1 [file EVA-14-566-s001.docx]
